# Universality, Temporality, and the Invisible Suffering of Menopause: A Philosophical Perspective in Psychiatry

**DOI:** 10.1192/j.eurpsy.2026.11897

**Published:** 2026-07-31

**Authors:** G. J. Cowen, J. French, M. M. Le, D. Nolasco, B. R. Carr

**Affiliations:** 1 School of Medicine, Wayne State University, Detroit, MI; 2 College of Medicine; 3 Department of Psychiatry, University of Florida, Gainesville, FL; 4 Department of Psychiatry and Behavioral Sciences, Stanford University, Stanford, CA, United States

## Abstract

**Introduction:**

Psychiatry often orients around pathological deviation from normative life stages. Menopause challenges this framework: a universal, predictable transition that nonetheless generates profound psychiatric vulnerability. This raises questions about psychiatry’s conceptual boundaries — how suffering is defined, when universality shields conditions from recognition, and how temporality shapes psychiatric risk.

**Objectives:**

To explore the philosophical dimensions of menopause in psychiatry, with emphasis on (1) universality versus pathology, (2) temporality and psychiatric risk, and (3) the ethics of invisibility in women’s midlife suffering.

**Methods:**

We conducted a focused literature review spanning philosophy of psychiatry, feminist theory, and phenomenology of time. Sources included theoretical texts on universality, stage transitions, and recognition, as well as psychiatric and philosophical scholarship addressing menopause and midlife suffering.

**Results:**

Three philosophical tensions emerged. (1) Universality versus pathology: Because menopause is normative, its psychiatric sequelae are often excluded from nosological frameworks, revealing a bias toward deviation rather than transition. (2) Temporality: Menopause highlights psychiatry’s difficulty conceptualizing time, since risk emerges not from disorder alone but from a predictable biological threshold—paralleling how PMDD ties affective change to cyclical temporality. (3) Invisibility of suffering: The under-recognition of menopause in psychiatric discourse perpetuates structural neglect, leaving women’s suffering conceptually and clinically unacknowledged.

**Conclusions:**

Menopause demonstrates psychiatry’s need to reconceptualize universality and temporality within its frameworks of suffering. Recognizing menopause as both a natural transition and a psychiatric risk state unveils the invisibility of women’s suffering, challenging psychiatry to reframe its philosophical boundaries. Such engagement would advance psychiatry not only clinically, but conceptually, by foregrounding life transitions as central to mental health.

**Disclosure of Interest:**

None Declared

